# Supplementary material for: Relationship between the evaluation of agricultural scientific and technological innovation capacity and the influencing factors of green agriculture
Source: PLoS One. 2023 Nov 30;18(11):e0295281. doi: 10.1371/journal.pone.0295281 (PMC10688882; doi:10.1371/journal.pone.0295281)
Supplement: Attachment — Submitted filename: Response to Reviewers.docx [file pone.0295281.s001.docx]

Reviewer #1:

1. In the study of green agriculture, the carbon footprint value is taken as the measurement index, and only the stock index is analyzed. The analysis of incremental data may produce new findings and results, so it is suggested to add this part of the study.--Added.
2. The literature review cited sufficient previous studies as the basis, but some of the references were blunt, so it was too natural to highlight the problems to be explained and the proof content of the references.--Modified.
3. If the data specifications in the text are inconsistent, the specifications should be unified and the accuracy should be consistent.--Modified.
4. As for research methods and data, this study lacks literature annotation on the source of methods and theoretical support.--Added.
5. This paper divides Guangdong Province into four parts: the Pearl River Delta region, the East wing region, the West wing region and the mountainous region. The prefecture-level cities included in each part should be indicated in the paper.--Indicated.
6. Guangdong Province is mostly hilly and mountainous. For the Pearl River Delta region, the eastern region, the western region and the mountainous region, their topographic characteristics and farming conditions are quite different, which should be taken into account when analyzing the differences.--Modified.
7. In the statistical relationship between variables tested by Granger causality test, changes in the carbon footprint of pesticides and fertilizers affect the degree of application of scientific and technological innovation in the agricultural field, while irrigation is not the cause of agricultural scientific and technological innovation, which indicates that the scientific and technological innovation ability of Guangdong Province in irrigation and water-saving needs to be further improved. In this paper, the analysis results of machinery, pesticides, fertilizers and irrigation should be correlated with the actual situation, and the results of data analysis should be verified by facts.--Modified.
8. In the impulse response function analysis of the impact effect of endogenous variables, the correlation between diesel consumption and agricultural science and technology should take into account not only the use of machinery, but also the use of new mechanical structures and the improvement of mechanical efficiency.--Modified.

Reviewer #2:

1. The introduction introduces the theme from the relevant situation of the current development of green agriculture and the progress of agricultural science and technology, and the respective is appropriate. The introduction content is relatively rich, however, the internal logic and relevance should be strengthened to explain the practical significance of carrying out this research.--Modified.
2. In this paper, some data are not labeled with reference to sources, such as the carbon footprint coefficient of green agricultural indicators such as diesel fuel, irrigation, fertilizer and pesticide.--Added.
3. This study analyzed the influencing factors of agricultural science and technology innovation ability and green agriculture, and took the contribution rate of agricultural science and technology progress as the representative of agricultural science and technology innovation ability and the carbon footprint as the representative of green agriculture, which has a certain theoretical basis. However, in the data analysis, conclusion and strategy part of the paper, the correlation between them is not fully discussed. It proves its correlation and practical significance more deeply.--Modified.
4. Broad agriculture includes agriculture (planting industry), forestry, animal husbandry, sideline industry and fishery (aquaculture industry). In this study, carbon emission indicators are mainly selected as machinery, irrigation, fertilizer and pesticide, which mainly refers to narrow agriculture and should be indicated in the paper.--Indicated narrow agriculture in the paper.
5. There are many factors affecting green agriculture. This paper chooses machinery, irrigation, fertilizer and pesticides as the main indicators. Although these four are the main sources of carbon emission, there lacks basis for selection.--Modified.
6. This paper takes the overall changes of the past five years as the evaluation node, corresponding to the major strategic stages of China's the 11th Five-Year Plan, the 12th Five-Year Plan and the 13th Five-Year Plan, which is highly representative. However, a brief introduction should be made to these stages to show their importance to scientific and technological progress, so as to strengthen the logic of the discussion.--Added.
7. There is a time lag between the innovation of agricultural research and its actual effect, which should be reflected in the relevant conclusions and discussions.--Modified.
8. In China's actual national conditions, agricultural production is changing from intensive cultivation of small farmers to mechanization and scale, and the input of farm manure and manpower in small farmer production is huge and difficult to measure, and there are some errors in statistical data, which can be taken into account in the study.--Modified.
